# Clinical characteristics of plastic bronchitis in children: a retrospective analysis of 43 cases

**DOI:** 10.1186/s12931-022-01975-1

**Published:** 2022-03-06

**Authors:** Jing-jing Huang, Xiao-qing Yang, Zhi-qiang Zhuo, Lin Yuan

**Affiliations:** 1 Third ward, Xiamen Children’s Hospital/Children’s Hospital of Fudan University Xiamen Branch, Xiamen, 361006 Fujian China; 2 Pediatrics Department, Women and Children’s Hospital, School of Medicine, Xiamen University/Xiamen Maternal and Child Health Care Hospital, Xiamen, 361003 Fujian China

**Keywords:** Plastic bronchitis, Children, Bronchoscopy

## Abstract

**Background:**

With an increase in the diagnosis of plastic bronchitis (PB) cases, to enhance paediatricians’ knowledge and add to the few existing studies, we explored the clinical characteristics, diagnosis, and treatment of PB in children.

**Methods:**

The clinicopathological data of 43 children admitted to the Xiamen Children’s Hospital and the Women and Children’s Hospital, affiliated to the Xiamen University from December 2016 to December 2019, were retrospectively analysed.

**Results:**

All the children had cough, with 41 of them having associated fever. A peak temperature > 40 ℃ was observed in 25 children. Twenty-six children had shortness of breath, 27 had reduced respiratory sounds on the affected side, and 35 had audible moist rales on the affected side. Lactate dehydrogenase in all children increased to different degrees, and 29 had elevated D-dimer and fibrinogen degradation products. Lung imaging showed pulmonary consolidation and atelectasis, mainly in the bilateral lower lung lobes, in all the children. However, 31 had pleural effusion, mainly a small parapneumonic effusion. The infections were mainly caused by adenovirus and *Mycoplasma pneumoniae*. The casts in all 43 children were sucked or clamped out under bronchoscopy, and 10 were found to have type I PB on pathological examination. All children were treated with anti-infective therapy in addition to bronchoscopic cast removal. Thirty-one children were treated with methylprednisolone, and 16 with gamma globulin. Except for one child who was non-adherent to treatment, all other children showed improvement, or were cured and discharged from the hospital. Follow-up lung imaging at 3 months revealed that the lungs were fully re-expanded in 40 children. At the 6-month follow-up, six children had small airway lesions, four had obliterative bronchiolitis, and one had bronchiectasis.

**Conclusions:**

Paediatric PB often occurs secondary to respiratory tract infections and progresses rapidly, with hyperpyrexia, cough, and shortness of breath as the main clinical manifestations. Pulmonary consolidation, atelectasis, and pleural effusion are seen on lung imaging, and early bronchoscopy and removal of casts in the trachea and bronchi are effective treatment options.

## Background

Plastic bronchitis (PB) is a rare respiratory emergency in paediatric patients. It is caused by endogenous tree-like casts or a jelly-like viscous substance blocking the trachea and bronchi, leading to pulmonary consolidation and atelectasis, and resulting in partial or complete pulmonary ventilation dysfunction and respiratory distress [1–3]. If the obstruction is not identified and eliminated in time, the disease can aggravate progressively, and can be even life-threatening in severe cases [4–6]. There are few previous reports on this disease. However, with the popularisation of bronchoscopy in recent years, more and more confirmed cases have been identified and cured. This study reviewed and summarised the clinical features, treatment, and prognosis of 43 children with PB to improve the understanding of PB among paediatricians.

## Methods

### Study design and setting

A total of 43 children with PB were included from December 2016 to December 2019, including 37 cases admitted in the Xiamen Children’s Hospital and 6 cases admitted in the Women and Children’s Hospital, affiliated to the Xiamen University. Patient medical history was collected after the parents provided written informed consent. This study was approved by the medical ethics committees of the hospitals.

### Diagnostic criteria

PB was diagnosed mainly using fibreoptic bronchoscopy and suctioning or clamping of tree-like bronchial casts [1, 2].

### Pathogen detection method

Diagnosis of ADV infection confirmed by nasopharyngeal swab or alveolar lavage fluid in children with seven respiratory viral antigen test (D3 Ultra DFA Respiratory Virus Screening, DH INC, China, Shanghai) or mNGS test (BGI.DX, China, Shenzhen). The diagnosis of mycoplasma pneumoniae was based on IgM detection of mycoplasma antibody (ELISA, FUJIREBIO INC, China, Zhuhai). Bacterial infection depends on culturing of blood, sputum, or alveolar lavage fluid (BD BACTEC FX, China, Xiamen). Fungal infection was based on the G or GM test (PlateliaTM Aspergillus Ag, A.C. Bio, China, Zhanjiang).

## Results

### Demographic characteristics

We included 24 boys and 19 girls, with a male-to-female ratio of 1.26:1. The age range was 7 months to 10 years, including 18 children (41.86%) aged ≤ 3 years, 19 children (44.19%) aged 4–6 years and 6 cases (13.95%) aged ≥ 7 years. Except for three children with underlying diseases (two had congenital heart diseases: atrial septal defect and ventricular septal defect, respectively; one had a history of recurrent respiratory tract infections), the other 40 children were previously in good health.

### Clinical manifestations and signs

The main clinical features of 43 children with PB were shown in Table 1.

**Table 1:** Clinical characteristics of 43 children with plastic bronchitis

Age (years)	n		Gender (M/F)		Fever time (d)	Discover PB time (d)	Dyspnea (%)	Pleural effusion (%)
≦3	18		9/9 (1:1)		7.50 ± 4.60	9.39 ± 3.62	10 (55.6%)	14 (77.8%)
4–6	19		10/9 (1.11:1)		8.39 ± 3.78	10.28 ± 4.48	5 (26.3%)	13 (68.4%)
≧7	6		5/1 (5:1)		8.14 ± 1.21	8.86 ± 2.27	1 (16.7%)	4 (66.7%)
Total	43		24/19 (1.26:1)		7.98 ± 3.84	9.67 ± 3.81	16 (37.3%)	31 (72.1%)

Age (years)	n	Electrolyte disturbance (%)	Abnormal myocardial enzyme (%)	Abnormal coagulation (%)	LDH *M (p25, p75)*
≦3	18	4 (22.2%)	5 (27.8%)	11 (61.1%)	635.5 (416.3, 1177.8)
4–6	19	5 (26.3%)	2 (10.5%)	12 (63.2%)	517.5 (327.7, 765)
≧7	6	2 (33.3%)	1 (16.7%)	4 (66.7%)	373 (291, 924)
Total	43	11 (25.6%)	8 (18.6%)	27 (62.8%)	533 (373, 918)

*PB* plastic bronchitis; *LDH* lactate dehydrogenase

All 43 children presented with cough, including 28 with cough and phlegm, and 15 with dry cough. A total of 41 children (95.3%) had fever, with 25 (58.1%) having a peak temperature > 40 ℃. The fever lasted 2–18 days. At the time of admission, 26 children (60.4%) had shortness of breath, among whom 12 had significant dyspnoea and hypoxaemia, and required oxygen support (including nine receiving nasal cannula oxygen therapy, two receiving high-flow oxygen therapy, and one receiving ventilator-assisted respiratory support). At the time of admission, 27 children (62.7%) had reduced respiratory sounds, moist rales could be heard on the affected side in 35 children (81.3%), and wheezing could be heard on the affected side in 13 children (30.2%).

### Etiological examination

Pathogens in 43 children with PB were shown in Table 2.

**Table 2:** Pathogens in 43 children with plastic bronchitis

Pathogens	n	%
MP	12	27.9
ADV	8	18.6
ADV + MP	7	16.3
influenza virus A and B	3	7
ADV + Aspergillus	2	4.7
ADV + Streptococcus pneumoniae	1	2.3
ADV + Haemophilus	1	2.3
MP + Moraxella catarrhalis	1	2.3
MP + Haemophilus	1	2.3
Haemophilus	1	2.3
*Streptococcus pneumoniae*	1	2.3
*Moraxella catarrhalis*	1	2.3
RSV	1	2.3
accidentally inhaled engine oil	1	2.3
no find	2	4.7

*ADV* adenovirus; *MP* *Mycoplasma pneumoniae*; *RSV* respiratory syncytial virus

Blood culture, sputum culture, immunofluorescence detection of seven respiratory viruses, *Mycoplasma pneumoniae* IgM and IgG serological tests, etc. were performed for all cases. Among them, 14 children had *M. pneumoniae* (MP) infection (including 2 with complicated bacterial infections, namely *Moraxella catarrhalis* and *Haemophilus influenzae*); 19 children had adenovirus (ADV) infection (including seven with complicated MP infection, two with complicated *Aspergillus* infection, one with complicated *H. influenzae* infection, and one with complicated *Streptococcus pneumoniae* infection); three children had influenza virus infection (including two with influenza A virus and one with influenza B virus infection); three children had bacterial infection (including one with *H. influenzae*, one with *Streptococcus pneumoniae*, and one with *M. catarrhalis* infection); one child had respiratory syncytial virus (RSV) infection; two children were infected with unknown pathogenic bacteria; and one child had accidentally inhaled engine oil.

### Laboratory examination

Among all the children, 10 had elevated peripheral blood leucocytes (> 10 × 10^9^/L); 27 had elevated C-reactive protein (CRP, > 8 mg/L; including 15 children with ADV infection, 12 with MP infection, and 7 infected with other pathogenic bacteria). Except for one child with ADV and *H. influenzae* infection with a CRP of 144.4 mg/L, all the other children had a CRP less than 45 mg/L. Twenty-three children had elevated procalcitonin (PCT, > 0.5 ng/mL, including 16 children with ADV infection, 3 with MP infection, 1 with influenza virus infection, 2 with bacterial infections, and 1 infected with unknown pathogenic bacteria). Lactate dehydrogenase (LDH) was increased in all the children, ranging from 252 to 2111 U/L (reference range: 114–240 U/L). D-dimer and fibrinogen degradation products (FDP) were increased in 29 children (67.4%).

### Imaging data

**Fig. 1:**
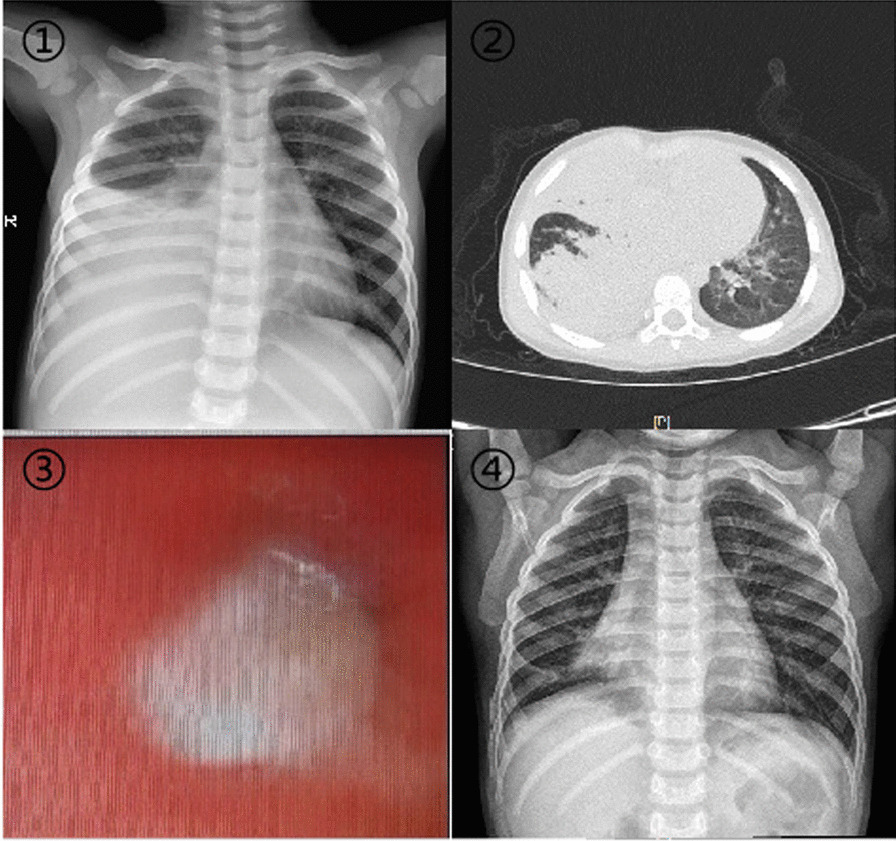
A case of plastic bronchitis caused by adenovirus infection. 1: Chest radiograph on admission: Pulmonary consolidation and atelectasis in the middle and lower lobes of the right lung. 2: Lung computed tomography on the second day after admission: Pulmonary consolidation and atelectasis in the middle and lower lobes of the right lung. 3: An obstructed lung lobe under bronchoscopy. 4: Chest radiograph on discharge: The pulmonary consolidation and atelectasis are resorbed

Chest radiography or computed tomography (CT) in all children showed segmental pulmonary consolidation and atelectasis (Fig. 1), including 29 with single-lobe lesions and 14 with lesions in ≥ 2 lung lobes; 32 had lesions in both lower lung lobes, and 11 had lesions involving the middle and upper lung lobes. A total of 31 children (72%) had pleural effusion. Except for 3 children undergoing thoracocentesis for moderate to large pleural effusion, all the other children had a small pleural effusion on the affected side.

### Bronchoscopic manifestations and pathological results

**Fig. 2:**
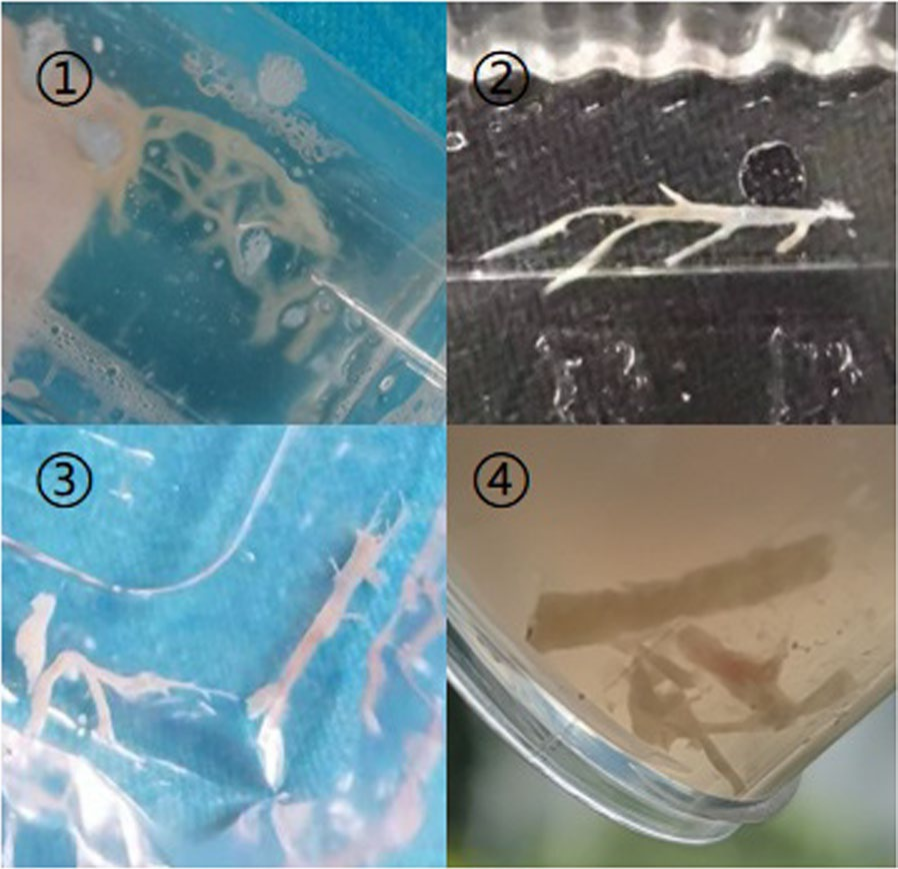
Display of various removed casts

**Fig. 3:**
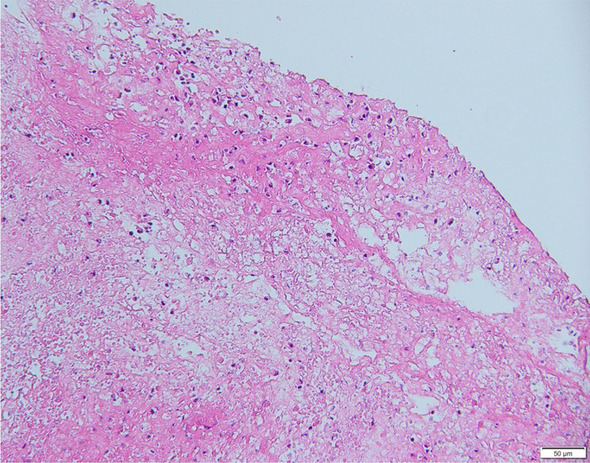
Pathological examination of casts: infiltration by a large number of neutrophils, and necrosis and exfoliation of the epithelial cells

Upon admission, all 43 children underwent bronchoscopy and bronchoalveolar lavage. In all cases, bronchial mucosa showed hyperaemia and oedema under bronchoscopy, and blockage by tree-like sputum bolts could be observed (Fig. 1). PB was diagnosed according to the plastic secretions removed by biopsy forceps or foreign body forceps (Fig. 2). Among these children, 20 had obstruction on the right side, 17 on the left side and 6 on the both sides (shown in Table 3). Clamping of casts and lavage under bronchoscopy were performed thrice in 3 children, twice in 17 children, and once in 23 children. Pathological examination was performed in 10 children (Fig. 3), revealing a large amount of celluloid necrotic substances, exfoliated epithelial cells, and inflammatory cell infiltration (mainly neutrophils and eosinophils) in all cases.

**Table 3:** Plastic casts involved sites in 43 cases of plastic bronchitis

PB involved lung lobes	n	%
left lung lobe	17	39.5
right lung lobe	20	46.5
both lung lobes	6	14
Total	43	100

*PB* plastic bronchitis

### Treatments and outcomes

During hospitalisation, all children were treated with anti-infective drugs, aerosol inhalation, phlegm resolution, and mechanical vibration sputum elimination in addition to cast removal and bronchoalveolar lavage under bronchoscopy. In addition, 31 children were treated with methylprednisolone (including 17 with ADV infection, 10 with MP infection, 3 with influenza virus infection, and 1 with RSV infection), and 16 children were treated with gamma globulin (including 14 with ADV infection, 1 with MP infection, and 1 with influenza virus infection). One child was treated with ventilator-assisted respiratory support, anti-infective drugs, methylprednisolone, gamma globulin, etc., and underwent cast removal through fibreoptic bronchoscopy after admission. However, the dyspnoea still had not improved significantly, and multiple organ failure occurred. The family members abandoned the child’s treatment, and the child had died according to telephone follow-up. All the other 42 children were discharged from the hospital after a median length of hospital stay of 12 (7–25) days. At the 3-month follow-up, lung imaging revealed that the lungs were re-expanded in 40 children. At the 6-month follow-up, six children had small airway lesions, four had obliterative bronchiolitis, and one had bronchiectasis.

## Discussion

The term “plastic bronchitis” originated as the endogenous foreign bodies in bronchi taken out under fibreoptic bronchoscopy are casts resembling the bronchial trees. Presently, the PB pathological classification proposed by Seear et al., which classifies PB into two types is still in use [7]. Type I (inflammatory type) is mainly characterised by a large amount of inflammatory cells (mainly neutrophils and eosinophils) and cellulose. Type II (non-inflammatory type) is mainly characterised by mucin and cellulose, mostly occurring after congenital heart disease repair, especially in children after the Fontan procedure [8]. In recent years, some scholars have found that malformations of pulmonary blood and lymphatic vessels can also lead to type II PB [1]. Presently, type I PB is considered to be closely associated with respiratory diseases, especially respiratory infections. In this study, all 43 children had an acute disease onset and showed respiratory system infections. In most of the cases, the infection sources were identified as viruses, mycoplasmas, or bacteria; therefore, they were presumed to be type I PB (i.e. inflammatory PB). Pathological examination of cast specimens was performed for 10 children, with the results suggesting type I PB, which also confirmed our judgement. There have also been similar reports from China and other countries. The reported common pathogens causing type I PB include *M. pneumoniae*, influenza virus, Epstein-Barr virus, and a few bacteria, etc. [4, 5, 9, 10]. The major pathogens vary in different regions and those in this study were adenovirus and *M. pneumoniae*. This is considered to be related to the different epidemiological trends of pathogens in different regions. In this study, adenoviral infection accounted for 44.1% of the cases, which is considered to be closely associated with the adenovirus epidemic in southern China in 2019. A case of PB was caused by accidental inhalation of engine oil. No pathological examination was done in this case, and it was speculated that engine oil caused chemical damage to the airway, leading to the release of a large number of inflammatory cells and even secondary infection. Therefore, the possibility of type I PB (i.e. inflammatory PB) was high.

The cases in this study included 24 boys and 19 girls, with a male-to-female ratio of 1.26:1, showing that the proportion of boys was slightly higher than that of girls. This is greatly different from the male-to-female ratios of 4:1 reported by Soyer et al. [11] and 5.3:1 by Sun et al. [12]. However, considering the small sample size, it cannot be concluded whether the male sex is a high-risk factor for PB, and further verification with large sample data is required. The ages at disease onset in the cases in this study ranged from 7 months to 10 years, but the median onset ages of PB caused by different pathogens were quite different. PB caused by ADV infection was more common in children aged under 2 years, while PB caused by MP infection mostly occurred in older children. Owing to the small numbers of cases infected by influenza virus and bacteria, statistical analysis could not be conducted. The difference in age at onset is considered to be related to the clinical characteristics of respiratory tract infections caused by different pathogens [13]. The time of finding the casts since disease onset in varied greatly, ranging from 2 to 21 days, but the casts were found within 1 week of onset in 19 cases, suggesting that the development and progression of this disease can be relatively fast. Children with PB usually show persistent hyperpyrexia, cough, shortness of breath, and other clinical manifestations. In this study, all 43 children had an acute disease onset with cough accompanied by fever as the main manifestation. In most of the children, reduced respiratory sounds and moist rales could be heard during lung auscultation. Among them, 26 children had complicated shortness of breath, 12 children had significant dyspnoea and hypoxaemia, and 17 children had stable respiration, which is considered to be related to the positions and degrees of obstruction of the airway by the casts. In severe cases, children with PB may also experience life-threatening multiple organ failure such as progressive respiratory failure and toxic encephalopathy. In this study, one child was administered routine treatment such as anti-infection after admission. On the third day after admission, bronchoalveolar lavage and cast removal were performed under fibreoptic bronchoscopy. Methylprednisolone and gamma globulin were added to the regimen. The condition still had not improved significantly, and dyspnoea was progressively aggravated. Multiple organ failure such as respiratory failure, toxic encephalopathy, and liver failure occurred. Therefore, the patient’s family decided to abandon treatment.

On laboratory examination, only 23.2% of the cases in this study had elevated white blood cells, all less than 15 × 10^9^/L. However, the proportions of cases with elevated CRP and PCT increased significantly, accounting for 62.7% and 53.8%, respectively. Therefore, the inflammatory reactions in children with PB was considered to be relatively serious. In addition, all 43 children had different degrees of LDH elevation. LDH, a cytoplasmic enzyme exists in various important organs such as the myocardium, liver, skeletal muscles, and lungs. For many diseases, the LDH level is a reliable index for judging disease severity and prognosis [14], and LDH elevation indicates that lysing of lung tissue or cell membrane damage [15]. In this study, 29 children (67.4%) had elevated D-dimer and FDP. Especially in children infected with ADV and MP, the hypercoagulable state can enhance bronchial cast formation, and physicians should be alert in this regard. D-dimer and FDP are degradation products of fibrin, generated under the action of plasmin. They are important indexes reflecting disorders of coagulation function. A study showed that the levels of plasma D-dimer and FDP can predict the severity and prognosis of community-acquired pneumonia and are closely correlated with the severity of pneumonia [16]. Therefore, we speculate that vascular endothelial cells are damaged during respiratory tract infections, exposing subcutaneous collagen and activating the coagulation system, resulting in a hypercoagulable state of blood, formation of microthrombi, and decline of blood flow permeability. These lead to reduced ventilation and air exchange capacity of lung tissue, retention of carbon dioxide, and changes in microcirculation of the lung tissue, causing retention of inflammatory factors and cells in the lungs, increased oozing of mucus, and formation of mucus plugs or casts.

Blockage by bronchial casts can lead to pulmonary consolidation and atelectasis. In this study, the lesions involved both lower lung lobes in 74.4% of the cases. It is speculated that casts are more likely to block both lower lung lobes, which is related to the patients’ positions and difficulty in clearing sputum from both lower lung lobes. In addition, among the cases in this study, 72% were complicated by pleural effusion, and most of them had a small amount of effusion on the affected side. The effusion could be absorbed spontaneously with treatment, suggesting that pulmonary consolidation and atelectasis as well as pleural effusion may be a major clinical feature of PB caused by respiratory tract infections [4, 17].

Sixteen children in this study had shortness of breath at the time of admission, with significant dyspnoea in 12 children. After treatment with anti-infectives and oxygen inhalation after admission, 16 children still had unsatisfactory improvement in shortness of breath. After removing the secretions and bronchial casts, the shortness of breath was significantly relieved, indicating that sucking or clamping out casts under fibreoptic bronchoscopy is the most important means to improve ventilation in children with PB [1, 18]. We suggest that the casts can cause severe or extensive airway blockage in some children, and long-term bronchoscopic operations may aggravate hypoxia; therefore, it is difficult to remove the casts completely at once. In such situations, drugs such as acetylcysteine or Mucosolvan can be used for lavage to promote dissolution of the casts, and foreign body forceps can be used repeatedly until the casts are successfully clamped out. In three children in this study, clamping under bronchoscopy was performed thrice before the casts were completely removed. Thirty-eight children still required drugs like anti-infectives, glucocorticoids, and gamma globulin to improve their clinical symptoms, which suggests that besides early identification and removal of bronchial casts to relieve respiratory tract obstruction, treatment of the primary disease is also equally important.

The gold standard for the diagnosis of PB is the removal of bronchial tree molding, which depends on bronchoscopy. At present, we advocate early bronchoscopy for children with radiographic findings of large patches of lung consolidation or atelectasis, which provides us with the possibility of finding plastic shape. As can be seen from the cases included in our study, the children had high fever accompanied by cough and dyspnea, and imaging showed atelectasis and abnormal coagulation function, which required high vigilance for the formation of plastic. Some scholars point out that the following situations also need to be alert to the occurrence of PB [3, 11, 18]: (1) Severe respiratory tract obstruction, ventilation dysfunction and intractable hypoxemia appeared in a short time. (2) Routine ventilation through endotracheal intubation ventilator, intensive nursing and sputum aspiration could not improve ventilation. (3) There was no obvious history of foreign body inhalation. Respiratory sound of lungs decreased or even disappeared.

### Limitations of this study

First, this is a retrospective analysis, and the summary of clinical characteristics of PB in children is still too general; In addition, no further studies have been done on the correlation between bronchoscopic manifestations of PB and etiology. Second, the sample size is still small, and the clinical features summarized in this paper may be limited. Third, 86% of all PB cases in this study had positive results of ADV and/or MP test. Compared with other pathogen infected cases, the difference in sample number was too significant to be suitable for inter-group statistical control study. Analysis of the reasons may be related to regional differences, epidemiological characteristics in recent two years and other factors, so the study results may be biased, and can not fully reflect the proportion of ADV and MP in PB infection pathogens.

## Conclusion

In conclusion, in children presenting with recurrent fever, shortness of breath, increased LDH, D-dimer, and FDP levels, and pulmonary consolidation and atelectasis accompanied by pleural effusion on lung imaging, PB should be suspected as a complication of an ongoing respiratory tract infection. We suggest that bronchoscopy should be performed as early as possible to remove the casts and relieve the respiratory tract obstruction. Meanwhile, attention should be paid to treatment of the primary disease in such cases.

## Data Availability

All data generated during this study are included in this published article.
